# A critical appraisal of the quality of adult dual-energy X-ray absorptiometry guidelines in osteoporosis using the AGREE II tool: An EuroAIM initiative

**DOI:** 10.1007/s13244-017-0553-6

**Published:** 2017-04-21

**Authors:** Carmelo Messina, Bianca Bignotti, Alberto Bazzocchi, Catherine M. Phan, Alberto Tagliafico, Giuseppe Guglielmi, Francesco Sardanelli, Luca Maria Sconfienza

**Affiliations:** 1 Scuola di Specializzazione in Radiodiagnostica, Università degli Studi di Milano, Via Festa del Perdono 7, 20122 Milan, Italy; 2 Department of Health Sciences, University of Genova, Genoa, Italy; 3 Diagnostic and Interventional Radiology, The “Rizzoli” Orthopaedic Institute, Via G. C. Pupilli 1, 40136 Bologna, Italy; 4 Department of Radiology, Saint-Antoine Hospital, Paris, France; 5 University of Genova and AOU IRCCS San Martino IST, Genoa, Italy; 6 Department of Radiology, University of Foggia, Foggia, Italy; 7 Servizio di Radiologia, IRCCS Policlinico San Donato, Piazza Malan 1, 20097 San Donato Milanese, Italy; 8 Dipartimento di Scienze Biomediche per la Salute, Università degli Studi di Milano, Via Pascal 36, 20133 Milan, Italy; 9 Unità Operativa di Radiologia Diagnostica ed Interventistica, IRCCS Istituto Ortopedico Galeazzi, Via Riccardo Galeazzi 4, 20161 Milan, Italy

**Keywords:** Dual-energy X-ray absorptiometry, DXA, Guidelines, Agree, Evidence based medicine

## Abstract

**Objectives:**

Dual energy X-ray absorptiometry (DXA) is the most widely used technique to measure bone mineral density (BMD). Appropriate and accurate use of DXA is of great importance, and several guidelines have been developed in the last years. Our aim was to evaluate the quality of published guidelines on DXA for adults.

**Methods:**

Between June and July 2016 we conducted an online search for DXA guidelines, which were evaluated by four independent readers blinded to each other using the AGREE II instrument. A fifth independent reviewer calculated scores per each domain and agreement between reviewers’ scores.

**Results:**

Four out of 59 guidelines met inclusion criteria and were included. They were published between 2005 and 2014. Three out of four guidelines reached a high level of quality, having at least five domain scores higher than 60%. Domain 1 (Scope and Purpose) achieved the highest result (total score = 86.8 ± 3.7%). Domain 6 (Editorial Independence) had the lowest score (total score = 54.7 ± 12.5%). Interobserver agreement ranged from fair (0.230) to good (0.702).

**Conclusions:**

Overall, the quality of DXA guidelines is satisfactory when evaluated using the AGREE II instrument. The Editorial Independence domain was the most critical, thus deserving more attention when developing future guidelines.

**Main messages:**

• *Three of four guidelines on DXA had a high quality level (>60%)*.

• *Scope/purpose had the highest score (86.8 ± 3.7%)*.

• *Editorial Independence had the lowest score (54.7 ± 12.5%)*.

• *Interobserver agreement ranged from fair (0.230) to good (0.702)*.

**Electronic supplementary material:**

The online version of this article (doi:10.1007/s13244-017-0553-6) contains supplementary material, which is available to authorized users.

## Introduction

Osteoporosis is defined as a systemic skeletal disease characterised by low bone mass and microarchitectural deterioration of bone tissue, with a subsequent increase in bone fragility and susceptibility to fracture [1]. Instrumental diagnosis of osteoporosis relies on bone mineral measurements, which can be obtained in vivo using different densitometric techniques. Among these, dual energy X-ray absorptiometry (DXA) is the most widely used in clinical practice [2–4]. Advantages of DXA are the very low radiation dose administered to patients, its very good reproducibility, and the capability to provide bone mineral density (BMD) values at central sites that relate to fracture risk [3, 5]. Other available techniques include quantitative ultrasound (QUS) and quantitative computed tomography (QCT) [6].

Appropriate and accurate use of densitometric techniques is of great importance: bone mineral measurements provide not only diagnostic criteria but also prognostic information on fracture risk probability, and they are also used to monitor treated or untreated patient [6]. For this reason, several guidelines have been developed in the last years with a number of recommendations that include indications for BMD testing, which skeletal site to measure, how to interpret and report BMD results, and proper timing for follow-up [7–10]. These guidelines, typically issued by relevant medical societies or specialised working groups, play an important role in clinical practice: they provide valuable suggestions based on the highest level of evidence, which is usually achieved through a critical evaluation of systematically searched primary studies [11, 12]. Nevertheless, clinical guidelines may vary widely in quality; as a consequence, it is important to evaluate the methods on which a guideline was developed in order to be confident with its recommendations [13, 14]. To do this, different quality appraisal instruments have been developed for evaluating guidelines. Among these, the Appraisal of Guidelines for Research & Evaluation version II (AGREE II) is reported to be a reliable, internationally used and validated tool [15].

The European Network for the Assessment of Imaging in Medicine European Institute for Biomedical Imaging Research (EuroAIM) was initiated with the aim to increase the evidence for the rational use of imaging technology [12, 16]. Currently, EuroAIM focused its attention on the evaluation of guidelines in different fields of diagnostic imaging. Regarding musculoskeletal radiology, a conjoined project between EuroAIM and the European Society of Musculoskeletal Radiology (ESSR) was established. DXA and densitometric techniques were included among the topic of interests. Therefore, the aim of this study is to evaluate the quality of current guidelines on DXA for adults using the AGREE II quality assessment tool.

## Materials and methods

Between June and July 2016 we searched for DXA guidelines using PubMed, EMBASE, Google and the Wiley Online Library, using the following keywords: “dual energy X-ray absorptiometry”, “DXA”, “DEXA”, “bone densitometry”, “Guidelines”, “Official Positions”, “Osteoporosis” and their expansions. Once guidelines had been retrieved, their references were also screened for further papers to include. We excluded from the results of our search those papers that were not primarily focused on DXA, such as national/international osteoporosis guidelines in which DXA was briefly mentioned in the context of a more comprehensive disease evaluation. Inclusion criteria were as follows: guidelines issued by national and international medical societies; full-manuscript available in English; guidelines must mainly contain recommendation on DXA, irrespective of other densitometric techniques; guidelines must focus mainly on the adult population (age >18 years).

The evaluation of guideline quality was made using the AGREE II instrument through the official website dedicated online platform [15]. The AGREE II protocol consists of 23 different items organised in 6 domains: domain 1 = “Scope and Purpose” (items 1–3); domain 2 = “Stakeholder Involvement” (items 4–6); domain 3 = “Rigor of Development” (items 7–14); domain 4 = “Clarity of Presentation” (items 15–17); domain 5 = “Applicability” (items 18–21); domain 6 = “Editorial Independence”. These six domains are followed by two additional items (“Overall Assessment”), which includes “the rating of the overall quality of the guideline and whether the guideline would be recommended for use in practice”. Table 1 shows a detailed description of all AGREE II items [15].

**Table 1:** Summary of AGREE II structure and detailed list of items within each domain (from reference 15)

Domain 1. Scope and Purpose	
Item 1	The overall objective(s) of the guideline is (are) specifically described
Item 2	The health question(s) covered by the guideline is (are) specifically described
Item 3	The population (patients, public, etc.) to whom the guideline is meant to apply is specifically described
Domain 2: Stakeholder Involvement	
Item 4	The guideline development group includes individuals from all the relevant professional groups
Item 5	The views and preferences of the target population (patients, public, etc.) have been sought
Item 6	The target users of the guideline are clearly defined
Domain 3: Rigor of Development	
Item 7	Systematic methods were used to search for evidence
Item 8	The criteria for selecting the evidence are clearly described
Item 9	The strengths and limitations of the body of evidence are clearly described
Item 10	The methods for formulating the recommendations are clearly described
Item 11	The health benefits, side effects and risks have been considered in formulating the recommendations
Item 12	There is an explicit link between the recommendations and the supporting evidence
Item 13	The guideline has been externally reviewed by experts prior to its publication
Item 14	A procedure for updating the guideline is provided
Domain 4: Clarity of Presentation	
Item 15	The recommendations are specific and unambiguous
Item 16	The different options for management of the condition or health issue are clearly presented
Item 17	Key recommendations are easily identifiable
Domain 5: Applicability	
Item 18	The guideline describes facilitators and barriers to its application
Item 19	The guideline provides advice and/or tools on how the recommendations can be put into practice
Item 20	The potential resource implications of applying the recommendations have been considered
Item 21	The guideline presents monitoring and/or auditing criteria
Domain 6: Editorial Independence	
Item 22	The views of the funding body have not influenced the content of the guideline
Item 23	Competing interests of guideline development group members have been recorded and addressed

Four independent reviewers (CM, BB, AB, CMP) with 4 to 15 years’ experience in musculoskeletal radiology and scientific research scored each guideline. All reviewers were previously trained to use AGREE II rating system by means of the user manual that was available on the online platform; in addition, reviewers were asked to complete two online training tools specifically developed to assist users in effectively applying the instrument. According to instruction tool, each item was rated on a 7-point scale ranging from 1 (strongly disagree, which means that no relevant information is provided) to 7 (strongly agree, which means that the quality of reporting is exceptional). Final domain scores were calculated by summing up all item scores within the domain and by scaling the total as a percentage of the maximum possible score for that domain [15].

### Data analysis

For analysis purposes, the evaluations performed by the four reviewers were averaged, and the average of each domain is reported in the results. Agreement between reviewers’ scores was calculated using the intraclass correlation coefficient (ICC), defined as follows: <0.20, poor; 0.21–0.40, fair; 0.41–0.60, moderate; 0.61–0.80, good; 0.81–1.00, very good. As for previous studies, the overall quality of each guidelines was evaluated using a threshold of 60% for the final score of each domain [17, 18]. High quality was defined when 5 or more domains scored >60%, average quality when 3 or 4 domains scored >60% and low-quality when ≤2 domains scored >60%. In addition, the total score (expressed as mean ± standard deviation, SD) of guidelines and domains was calculated. Domain scores were categorised as good (≥80%), acceptable (60–79%), low (40–59%) or very low (<40%), similar to a previous similar paper [19]. Data collection, extraction and scoring were performed by a fifth independent reviewer (LMS) with 12 years’ experience in in musculoskeletal radiology and scientific research, using a Microsoft Excel® 2016 spreadsheet. ICC calculations were performed using the SPSS software (version 24, IBM, Armonk, NY).

## Results

Our strategy of literature search identified 59 guidelines, 4 of which met our inclusion criteria [7–10]. Exclusion of guidelines was mainly based on the following criteria: guidelines were primarily focused on a densitometric technique different from DXA, such as quantitative computed tomography; the main target was pediatric population; fracture risk assessment guidelines; body composition guidelines. The characteristics of DXA guidelines that were included in our study are presented in Table 2. Year of publication ranged from 2005 to 2014.

**Table 2:** General characteristics of DXA guidelines included in the analysis

DXA guideline title	Country of origin	Year of publication	Organisation
Recommendations for Bone Mineral Density Reporting in Canada [8]	Canada	2005	Canadian Association of Radiologists
International Society for Clinical Densitometry 2007 Adult and Pediatric Official Positions [7]	USA	2008	International Society for Clinical Densitometry
ACR Appropriateness Criteria: Osteoporosis and Bone Mineral Density [10]	USA	2010	American College of Radiology (ACR)
ACR-SPR-SSR Practice Parameter for the Performance of Dual-Energy X-Ray Absorptiometry [9]	USA	2014	American College of Radiology (ACR), Society for Pediatric Radiology (SPR), Society of Skeletal Radiology (SSR)

Table 3 summarises the total score for each domain as well as the final judgment of overall quality. Detailed scores and reviewers’ comments for each guideline are reported in Supplementary Tables 1, 2, 3 and 4. Three out of four guidelines reached a high level of quality, having at least five domain scores higher than 60%. Among these, “Adult and Pediatric Official Positions” issued by the International Society for Clinical Densitometry (ISCD) [7] achieved the highest total score (76.1%). The only guideline that reached an average level of quality was the one issued conjoinedly by the American College of Radiology (ACR), the Society for Pediatric Radiology (SPR) and the Society of Skeletal Radiology (SSR) [9], with only four domains scoring >60% and a total score of 64.1% ±11.3% [mean ± standard deviation (SD)]. ISCD Official Position was the guideline with the more variable scores, with a SD of 18.1%, while the Canadian recommendation paper [8] had the lowest variability (SD = 9.5%). Supplementary Tables 1, 2, 3 and 4 show the detailed AGREE II domain scores for each guideline.

**Table 3:** Summary of the average of domain scores of DXA Guidelines according to AGREE II

Guideline title	Domain 1	Domain 2	Domain 3	Domain 4	Domain 5	Domain 6	Total score mean (SD)	Overall quality
Recommendations for BMD Reporting in Canada [8]	84.7%* (good)	73.6%* (acceptable)	57.3% (low)	77.8%* (acceptable)	66.7%* (acceptable)	75.0%* (acceptable)	72.5% (9.5%)	High
ISCD 2007 Adult and Pediatric Official Positions [7]	91.7%* (good)	76.4%* (acceptable)	78.6%* (acceptable)	90.3%* (good)	78.1%* (acceptable)	41.7% (low)	76.1% (18,1%)	High
ACR Appropriateness Criteria: Osteoporosis and BMD [10]	88.9%* (good)	68.1%* (acceptable)	60.9%* (acceptable)	87.5%* (good)	76.0%* (acceptable)	54.2% (low)	72.6% (14.1%)	High
ACR-SPR-SSR Practice Parameter for the Performance of DXA [9]	81.9%* (good)	68.1%* (acceptable)	58.9% (low)	66.7%* (acceptable)	61.5%* (acceptable)	47.9% (low)	64.1% (11.3%)	Average
Total domain score; mean (SD)	86,8% (3,7%) (good)	71,5% (3.6%) (acceptable)	63.9% (8.6%) (acceptable)	80.6% (9.3%) (good)	70.6% (6.8%) (acceptable)	54.7% (12.5%) (low)		

Domain 1 = scope and purpose; domain 2 = stakeholder involvement; domain 3 = rigor of development; domain 4 = clarity of presentation; domain 5 = applicability; domain 6 = editorial independence. Domain scores ≥80% = good; 60–79% = acceptable; 40–59% = low; <40% = very low. * = total score of domain >60%. BMD = Bone mineral density; ISCD = International Society for Clinical Densitometry; ACR = American College of Radiology; SPR = Society for Pediatric Radiology; SSR = Society of Skeletal Radiology; SD = standard deviation

Domain scores ranged between 41.7% (lowest value, domain 6 of ISCD Official Positions) and 91.7% (highest value, domain 1 of ISCD Official Positions). When comparing the scores of each domain across guidelines, “Scope and Purpose” (domain 1) and “Clarity of Presentations” (domain 4) achieved the highest results, with a total domain score of 86.8 ± 3.7% and 80.6 ± 9.3%, respectively. The domain with the lowest total score was “Editorial Independence” (domain 6), with a total mean score of 54.7 ± 12.5%.

Total mean score of domain 1 (“Scope and Purpose”) was 86.8% with low variability (SD = 3.7%). The guideline published by ISCD reached the highest score (91.7% = good), while ACR-SPR-SSR conjoined guideline achieved a score of 81.9%, which is still considered “good”.

For domain 2 (“Stakeholder Involvement”), the overall mean score was “acceptable” with a mean score of 71.5%. Quality scores variability was low (SD = 3.6%). Again, ISCD Official Positions was the guideline with the highest score (76.4% = acceptable), while both ACR and ACR-SPR-SSR guidelines scored the lowest value (68.1% = acceptable).

Domain 3 (“Rigor of Development”) had the second-lowest mean score (63.9%) with a slightly higher variability (SD = 6.8%) compared to domain 1 and 2. ISCD Official Positions was the guideline with the highest score (78.6% = acceptable), while Canadian Guideline had the lowest score (57.3% = low).

Domain 4 (“Clarity of Presentation”) had the second-highest mean quality score (80.6%), with 9.3% SD. Guideline scores ranged from 90.3% (good) of ISCD Official Positions to 66.7% (acceptable) of the ACR-SPR-SSR Guideline.

Total mean score of domain 5 (“Applicability”) was 70.6% with intermediate variability (SD = 6.8%). Within this domain, ISCD had the highest score (78.1% = acceptable) while the ACR-SPR-SSR Guideline had the lowest (61.5% = acceptable).

The lowest scores were obtained by domain 6 (“Editorial Independence”), with a total mean score of 54.7%; this domain had also the larger variability, with 12.5% SD. The guideline published by the Canadian Association of Radiologists reached the better score (75% = acceptable); differently from the previous domain, the ISCD Official Positions had the lowest domain score (41.7% = low).

Interobserver variability ranges were 0.702 (good; 95% confidence interval, 0.438–0.860) for the ISCD guidelines, 0.230 (fair; −0.454-0.639) for the ACR-SPR-SSR guideline, 0.451 (moderate; −0.037-0.743) for the Canadian Association of Radiologists guideline and 0.474 (moderate; −0.006-0.753) for the ACR guideline.

## Discussion

Our main finding is that the AGREE II appraisal of the DXA guidelines showed satisfactory results as the overall quality was high in three out of four guidelines and that the domain score never decreased under 40%. However, a wide variability was found across the six domains, with scores that ranged from “good” to “low” in all guidelines. Results were somehow uniform when considering the within-domain scores; among these, domain 1 scored all “good” percentages with low variability, which means that the scope and purpose of all the evaluated guidelines was well described.

Domains with the highest quality were “Scope and Purpose” and “Clarity of Presentation”; both scored over 80%. This finding is comparable to different previous guideline evaluation studies with the AGREE II instrument, regardless of the topic [18–21]. The reason for such high scores regardless of the topic is not clear [18]. This may be attributable to the fact that both domains 1 and 3 contain fundamental elements that cannot be easily omitted, such as guideline objectives, the health question to deal with and the population to whom the guideline is applied.

Editorial independence (domain 6) scored low in all guidelines with the exception of the “Recommendations for BMD Reporting in Canada”. Thus, this was our poorest scoring domain (54.7%). Armstrong et al. reported similar results (45%) after conducting an evaluation of osteoporosis guidelines focusing on physical activity and safe movement [18]. This domain scored low in several other studies [19, 20, 22, 23], with few exceptions [21]. According to AGREE II, the evaluation of “editorial independence” considers two aspects related to funding bodies or potential authors’ competing interests that may influence the guideline content. An explicit statement that the funding body interests have not influenced the final recommendations should be present; at the same time, all guideline authors should provide a disclosure of all competing interests. This information is not reported clearly in these guidelines, in particular for ISCD Official Positions, a paper that scored very well for the remaining domains. This is a critical aspect, as it has been shown that conflicts of interest among authors of such guidelines are very common and may affect the quality of final recommendations [18, 24–26]. Therefore, high quality for this domain is particularly needed, especially for those guidelines with recommendations on diagnostic technologies or medications.

When considering the quality of DXA guidelines over time, we observed a decrease of the overall scores. The ACR-SPR-SSR guideline, published in 2014, had a score lower than 8.4% of the guideline issued in 2005 by the Canadian Association of Radiologists. This finding is in accordance with a review published in 2012 by Kung et al., which found no clear improvement of guideline quality over the past 2 decades [27]. Conversely, Armstrong et al. found quality improvement over time [18]. The limited number of studies we included in our review may perhaps explain our different results.

One issue of this analysis, which may be seen as a limitation, is that interobserver reproducibility was low, except for the ISCD guideline. Analysing the scores and the comments provided by the reviewers in detail, the highest variability was found for the Applicability and Editorial Independence domains. Regarding applicability, some reviewers found that information was not clearly presented, while others considered them implicit in the provided statements. Regarding Editorial Independence, we note that in most cases information about funding and competing interests were provided in documents/links separated from the main paper. Thus, some reviewers considered that the information was not present, while others browsed the additional documents to find it. These data mean that, despite previous training, reviewers had different interpretations of the same items: some were very adherent to what stated by the AGREE II, while others had a broader interpretation. Of note, a wide range of interobserver variability (0.34 to 0.65) was also reported in a previous paper that used the same tool to evaluate osteoporosis clinical practice guidelines for physical activity and safe movement [18].

Some limitations of this study are intrinsic to the AGREE II, as this instrument is not aimed to evaluate all aspects of a guideline. In particular, AGREE II does not evaluate the degree of consistency between the guideline recommendations and the reported evidence [19]. Also, AGREE II does not specifically evaluate the clinical content, a limitation that is common to several appraisal tools [28]. Then, the four reviewers of this work have different experience in DXA and guideline evaluation, potentially biasing the outcome. However, the use of average scores and previous training on the proper use of the AGREE II instrument may have reduced the impact of this limitation. Last, as mentioned above, the number of DXA guidelines included in the evaluation is small.

In conclusion, evidence-based guidelines are of vital importance to provide valuable suggestions to physicians in the daily clinical practice. Our study showed that the overall quality of the DXA guidelines is satisfactory according to the AGREE II evaluation instrument. The domain of “Editorial Independence” was the most critical one in terms of overall score; thus emphasis should be given to these aspects in order to provide unbiased recommendations. When developing future guidelines, authors should also take this domain into account as it may bring clinical consequences.

## Electronic supplementary material

Table S1 (DOCX 17 kb)

Table S2 (DOCX 17 kb)

Table S3 (DOCX 17 kb)

Table S4 (DOCX 17 kb)
