# Supplementary material for: A critical appraisal of the quality of adult dual-energy X-ray absorptiometry guidelines in osteoporosis using the AGREE II tool: An EuroAIM initiative
Source: Insights Imaging. 2017 Apr 21;8(3):311–7. doi: 10.1007/s13244-017-0553-6 (PMC5438319; doi:10.1007/s13244-017-0553-6)
Supplement: Supplementary file 1 — (DOCX 17 kb) [file 13244_2017_553_MOESM1_ESM.docx]

**Supplementary Table 1** Detailed AGREE II domain scores for the guideline “Recommendations for Bone Mineral Density Reporting in Canada” [8]

| **Domain** | **Item** | **Rater 1** | **Rater 2** | **Rater 3** | **Rater 4** | **Total** | **Total per Domain** | **Domain score** |
| --- | --- | --- | --- | --- | --- | --- | --- | --- |
| Scope and Purpose | ***1*** | 7 | 7 | 7 | 7 | 28 | 73 | **84,7%** |
|  | ***2*** | 7 | 5 | 7 | 3 | 22 |  |  |
|  | ***3*** | 7 | 6 | 5 | 5 | 23 |  |  |
| Stakeholder Involvement | ***4*** | 7 | 6 | 7 | 7 | 27 | 65 | **73,6%** |
|  | ***5*** | 7 | 4 | 3 | 2 | 16 |  |  |
|  | ***6*** | 6 | 6 | 7 | 3 | 22 |  |  |
| Rigour of Development | ***7*** | 7 | 6 | 1 | 3 | 17 | 142 | **57,3%** |
|  | ***8*** | 6 | 6 | 2 | 2 | 16 |  |  |
|  | ***9*** | 7 | 5 | 1 | 3 | 16 |  |  |
|  | ***10*** | 7 | 5 | 4 | 4 | 20 |  |  |
|  | ***11*** | 7 | 5 | 1 | 3 | 16 |  |  |
|  | ***12*** | 6 | 5 | 2 | 4 | 17 |  |  |
|  | ***13*** | 5 | 5 | 7 | 5 | 22 |  |  |
|  | ***14*** | 2 | 6 | 4 | 6 | 18 |  |  |
| Clarity of Presentation | ***15*** | 6 | 6 | 5 | 7 | 24 | 68 | **77,8%** |
|  | ***16*** | 6 | 5 | 2 | 6 | 19 |  |  |
|  | ***17*** | 7 | 6 | 5 | 7 | 25 |  |  |
| Applicability | ***18*** | 7 | 6 | 5 | 4 | 22 | 80 | **66,7%** |
|  | ***19*** | 7 | 6 | 6 | 5 | 24 |  |  |
|  | ***20*** | 2 | 3 | 5 | 3 | 13 |  |  |
|  | ***21*** | 6 | 5 | 7 | 3 | 21 |  |  |
| Editorial Independence | ***22*** | 6 | 7 | 7 | 7 | 27 | 44 | **75,0%** |
|  | ***23*** | 7 | 7 | 1 | 2 | 17 |  |  |
