# Supplementary material for: A critical appraisal of the quality of adult dual-energy X-ray absorptiometry guidelines in osteoporosis using the AGREE II tool: An EuroAIM initiative
Source: Insights Imaging. 2017 Apr 21;8(3):311–7. doi: 10.1007/s13244-017-0553-6 (PMC5438319; doi:10.1007/s13244-017-0553-6)
Supplement: Supplementary file 2 — (DOCX 17 kb) [file 13244_2017_553_MOESM2_ESM.docx]

**Supplementary Table 2** Detailed AGREE II domain scores for the guideline “ISCD 2007 Adult and Pediatric Official Positions” [7]

| **Domain** | **Item** | **Rater 1** | **Rater 2** | **Rater 3** | **Rater 4** | **Total** | **Total per Domain** | **Domain score** |
| --- | --- | --- | --- | --- | --- | --- | --- | --- |
| Scope and Purpose | ***1*** | 7 | 7 | 5 | 7 | 26 | 78 | **91,7%** |
|  | ***2*** | 6 | 7 | 6 | 6 | 25 |  |  |
|  | ***3*** | 7 | 7 | 6 | 7 | 27 |  |  |
| Stakeholder Involvement | ***4*** | 6 | 6 | 7 | 6 | 25 | 67 | **76,4%** |
|  | ***5*** | 6 | 6 | 5 | 2 | 19 |  |  |
|  | ***6*** | 6 | 7 | 4 | 6 | 23 |  |  |
| Rigour of Development | ***7*** | 7 | 7 | 5 | 7 | 26 | 183 | **78,6%** |
|  | ***8*** | 6 | 7 | 5 | 7 | 25 |  |  |
|  | ***9*** | 6 | 5 | 2 | 5 | 18 |  |  |
|  | ***10*** | 7 | 6 | 7 | 6 | 26 |  |  |
|  | ***11*** | 6 | 6 | 2 | 5 | 19 |  |  |
|  | ***12*** | 7 | 7 | 6 | 5 | 25 |  |  |
|  | ***13*** | 6 | 6 | 6 | 6 | 24 |  |  |
|  | ***14*** | 3 | 7 | 6 | 4 | 20 |  |  |
| Clarity of Presentation | ***15*** | 6 | 7 | 7 | 7 | 27 | 77 | **90,3%** |
|  | ***16*** | 6 | 7 | 4 | 6 | 23 |  |  |
|  | ***17*** | 7 | 6 | 7 | 7 | 27 |  |  |
| Applicability | ***18*** | 6 | 6 | 6 | 5 | 23 | 91 | **78,1%** |
|  | ***19*** | 6 | 6 | 6 | 5 | 23 |  |  |
|  | ***20*** | 7 | 6 | 5 | 4 | 22 |  |  |
|  | ***21*** | 7 | 6 | 7 | 3 | 23 |  |  |
| Editorial Independence | ***22*** | 6 | 7 | 1 | 6 | 20 | 28 | **41,7%** |
|  | ***23*** | 1 | 3 | 1 | 3 | 8 |  |  |
