# Supplementary material for: A critical appraisal of the quality of adult dual-energy X-ray absorptiometry guidelines in osteoporosis using the AGREE II tool: An EuroAIM initiative
Source: Insights Imaging. 2017 Apr 21;8(3):311–7. doi: 10.1007/s13244-017-0553-6 (PMC5438319; doi:10.1007/s13244-017-0553-6)
Supplement: Supplementary file 3 — (DOCX 17 kb) [file 13244_2017_553_MOESM3_ESM.docx]

**Supplementary Table 3** Detailed AGREE II domain scores for the guideline “ACR Appropriateness Criteria: Osteoporosis and BMD” [10]

| **Domain** | **Item** | **Rater 1** | **Rater 2** | **Rater 3** | **Rater 4** | **Total** | **Total per Domain** | **Domain score** |
| --- | --- | --- | --- | --- | --- | --- | --- | --- |
| Scope and Purpose | ***1*** | 7 | 6 | 6 | 6 | 25 | 76 | **88,9%** |
|  | ***2*** | 6 | 6 | 7 | 5 | 24 |  |  |
|  | ***3*** | 6 | 7 | 7 | 7 | 27 |  |  |
| Stakeholder Involvement | ***4*** | 5 | 5 | 5 | 4 | 19 | 61 | **68,1%** |
|  | ***5*** | 7 | 5 | 3 | 5 | 20 |  |  |
|  | ***6*** | 6 | 6 | 4 | 6 | 22 |  |  |
| Rigour of Development | ***7*** | 6 | 5 | 3 | 4 | 18 | 149 | **60,9%** |
|  | ***8*** | 5 | 4 | 2 | 5 | 16 |  |  |
|  | ***9*** | 6 | 5 | 3 | 6 | 20 |  |  |
|  | ***10*** | 6 | 5 | 2 | 6 | 19 |  |  |
|  | ***11*** | 6 | 6 | 5 | 6 | 23 |  |  |
|  | ***12*** | 6 | 5 | 5 | 6 | 22 |  |  |
|  | ***13*** | 6 | 4 | 2 | 4 | 16 |  |  |
|  | ***14*** | 2 | 5 | 1 | 7 | 15 |  |  |
| Clarity of Presentation | ***15*** | 6 | 6 | 7 | 7 | 26 | 75 | **87,5%** |
|  | ***16*** | 2 | 7 | 7 | 6 | 22 |  |  |
|  | ***17*** | 6 | 7 | 7 | 7 | 27 |  |  |
| Applicability | ***18*** | 6 | 7 | 6 | 6 | 25 | 89 | **76,0%** |
|  | ***19*** | 6 | 6 | 7 | 5 | 24 |  |  |
|  | ***20*** | 2 | 6 | 5 | 6 | 19 |  |  |
|  | ***21*** | 6 | 5 | 6 | 4 | 21 |  |  |
| Editorial Independence | ***22*** | 7 | 7 | 1 | 6 | 21 | 34 | **54,2%** |
|  | ***23*** | 6 | 2 | 1 | 4 | 13 |  |  |
