# Supplementary material for: A critical appraisal of the quality of adult dual-energy X-ray absorptiometry guidelines in osteoporosis using the AGREE II tool: An EuroAIM initiative
Source: Insights Imaging. 2017 Apr 21;8(3):311–7. doi: 10.1007/s13244-017-0553-6 (PMC5438319; doi:10.1007/s13244-017-0553-6)
Supplement: Supplementary file 4 — (DOCX 17 kb) [file 13244_2017_553_MOESM4_ESM.docx]

**Supplementary Table 4** Detailed AGREE II domain scores for the guideline “ACR–SPR–SSR Practice Parameter for the Performance of DXA” [9]

| **Domain** | **Item** | **Rater 1** | **Rater 2** | **Rater 3** | **Rater 4** | **Total** | **Total per Domain** | **Domain score** |
| --- | --- | --- | --- | --- | --- | --- | --- | --- |
| Scope and Purpose | ***1*** | 6 | 7 | 7 | 5 | 25 | 71 | **81,9%** |
|  | ***2*** | 4 | 6 | 7 | 4 | 21 |  |  |
|  | ***3*** | 6 | 6 | 7 | 6 | 25 |  |  |
| Stakeholder Involvement | ***4*** | 5 | 6 | 6 | 3 | 20 | 61 | **68,1%** |
|  | ***5*** | 6 | 4 | 4 | 2 | 16 |  |  |
|  | ***6*** | 6 | 7 | 7 | 5 | 25 |  |  |
| Rigour of Development | ***7*** | 6 | 6 | 3 | 3 | 18 | 145 | **58,9%** |
|  | ***8*** | 7 | 6 | 1 | 2 | 16 |  |  |
|  | ***9*** | 6 | 6 | 1 | 4 | 17 |  |  |
|  | ***10*** | 6 | 6 | 2 | 4 | 18 |  |  |
|  | ***11*** | 4 | 6 | 3 | 5 | 18 |  |  |
|  | ***12*** | 6 | 5 | 5 | 4 | 20 |  |  |
|  | ***13*** | 6 | 5 | 3 | 3 | 17 |  |  |
|  | ***14*** | 2 | 7 | 7 | 5 | 21 |  |  |
| Clarity of Presentation | ***15*** | 6 | 6 | 7 | 7 | 26 | 60 | **66,7%** |
|  | ***16*** | 2 | 7 | 2 | 3 | 14 |  |  |
|  | ***17*** | 6 | 5 | 7 | 2 | 20 |  |  |
| Applicability | ***18*** | 6 | 5 | 5 | 3 | 19 | 75 | **61,5%** |
|  | ***19*** | 6 | 6 | 7 | 3 | 22 |  |  |
|  | ***20*** | 6 | 2 | 3 | 3 | 14 |  |  |
|  | ***21*** | 5 | 6 | 5 | 4 | 20 |  |  |
| Editorial Independence | ***22*** | 7 | 7 | 1 | 2 | 17 | 31 | **47,9%** |
|  | ***23*** | 7 | 4 | 1 | 2 | 14 |  |  |
